# RNA-seq analysis and compound screening highlight multiple signalling pathways regulating secondary cell death after acute CNS injury *in vivo*

**DOI:** 10.1242/bio.050260

**Published:** 2020-05-04

**Authors:** Chiara Herzog, David Greenald, Juan Larraz, Marcus Keatinge, Leah Herrgen

**Affiliations:** Centre for Discovery Brain Sciences, Deanery of Biomedical Sciences, The University of Edinburgh, 49 Little France Crescent, Edinburgh EH16 4SB, UK

**Keywords:** CNS injury, Secondary cell death, RNA-seq, Compound screen, Cellular signalling, Zebrafish

## Abstract

Understanding the molecular mechanisms that regulate secondary cell death after acute central nervous system (CNS) injury is critical for the development of effective neuroprotective drugs. Previous research has shown that neurotoxic processes including excitotoxicity, oxidative stress and neuroinflammation can cause secondary cell death. Nevertheless, clinical trials targeting these processes have been largely unsuccessful, suggesting that the signalling pathways underlying secondary cell death remain incompletely understood. Due to their suitability for live imaging and their amenability to genetic and pharmacological manipulation, larval zebrafish provide an ideal platform for studying the regulation of secondary cell death *in vivo*. Here, we use RNA-seq gene expression profiling and compound screening to identify signalling pathways that regulate secondary cell death after acute neural injury in larval zebrafish. RNA-seq analysis of genes upregulated in cephalic *mpeg1^+^* macrophage-lineage cells isolated from *mpeg1:GFP* transgenic larvae after neural injury suggested an involvement of cytokine and polyamine signalling in secondary cell death. Furthermore, screening a library of FDA approved compounds indicated roles for GABA, serotonin and dopamine signalling. Overall, our results highlight multiple signalling pathways that regulate secondary cell death *in vivo*, and thus provide a starting point for the development of novel neuroprotective treatments for patients with CNS injury.

This article has an associated First Person interview with the two first authors of the paper.

## Abstract (precis)

**Summary:** Multiple cellular signalling pathways are involved in the regulation of secondary cell death after nervous system injury.

## INTRODUCTION

Acute central nervous system (CNS) injury causes the death of neural cells, which occurs in two phases. Primary cell death is the direct and immediate consequence of an acute insult such as physical trauma. In contrast, secondary cell death occurs in the minutes, hours and days after the initial insult as an indirect result of complex neurotoxic processes triggered by the primary injury. Secondary cell death plays an important role in the pathophysiology of acute CNS disorders such as traumatic brain injury (Loane et al., 2015; Park et al., 2008), spinal cord injury (Kwon et al., 2004; Oyinbo, 2011), and stroke (Xing et al., 2012; Yuan, 2009). Importantly, the delayed occurrence of secondary cell death opens a therapeutic window during which treatments aimed at reducing or preventing it could be applied. Accordingly, intense efforts have been made to identify the molecular and cellular mechanisms that regulate secondary cell death. This research has shown that cellular processes including excitotoxicity, oxidative stress and neuroinflammation can contribute to secondary cell death after acute neural injury.

Excitotoxicity causes neural cell death by excessive stimulation of glutamate receptors, whereas oxidative stress can kill cells through the toxic effects of reactive oxygen species. In addition, the inflammatory response to neural injury plays an important role in the regulation of secondary cell death. Neuroinflammation was long thought to be primarily detrimental, but there is an increasing realisation that it can also have neuroprotective effects (Loane and Kumar, 2016; Xiong et al., 2016; Zhou et al., 2014). Both brain-resident microglia and peripheral macrophages are important cellular effectors of the inflammatory response to neural injury. They migrate to the site of injury, where they remove dead cells and cellular debris through phagocytosis (Hanisch and Kettenmann, 2007; Nimmerjahn et al., 2005). Furthermore, microglia and macrophages respond to CNS injury by secreting a wide range of effector molecules including pro- and anti-inflammatory cytokines, chemokines and neurotrophic factors (Anwar et al., 2016; Hellewell et al., 2016; Mracsko and Veltkamp, 2014).

Despite great efforts to develop neuroprotective treatments for patients with acute CNS injury, clinical trials of drugs targeting excitotoxicity, oxidative stress or neuroinflammation after traumatic brain injury (Chakraborty et al., 2016; Hawryluk and Bullock, 2015; Maas et al., 2008), spinal cord injury (Ahuja et al., 2017; Kim et al., 2017) or stroke (Chamorro et al., 2016; Moretti et al., 2015) have largely failed. This indicates that the signalling pathways that regulate secondary cell death remain incompletely understood, and that more research is needed before effective drugs can be developed.

Due to their suitability for live imaging and their amenability to genetic and pharmacological manipulation, larval zebrafish provide an ideal platform for the rapid identification of genes and compounds that modulate secondary cell death *in vivo*. In previous work, we established an experimental setup for quantification of secondary cell death after acute CNS injury in larval zebrafish, and showed that neurotoxic processes such as excitotoxicity that underlie secondary cell death in mammals are conserved in this model system (Herzog et al., 2019). Here we utilise this platform, in combination with RNA-seq gene expression profiling and compound screening, to identify signalling pathways that modulate secondary cell death *in vivo*. First, we conduct RNA-seq analysis to identify signalling molecules secreted by microglia and macrophages after CNS injury in larval zebrafish. Our results reveal 17 such factors, including cytokines, chemokines, growth factors, and enzymes involved in polyamine and arachidonic acid metabolism. We then use CRISPR/Cas9-mediated gene knockout to test the involvement of a subset of these genes in injury-induced cell death, and find that knockout of the cytokine *tnfsf11* or the polyamine-metabolising enzyme *Smox* leads to an increase in secondary cell death. Second, we conduct an imaging-based screen of 786 FDA-approved compounds to identify small molecules that modulate secondary cell death *in vivo*. This revealed two compounds that consistently and specifically change injury-induced cell death. Of these, the GABA reuptake inhibitor tiagabine decreases cell death, whereas the serotonin and dopamine receptor antagonist ziprasidone increases it. Overall, these results indicate that a range of cellular signalling pathways are involved in regulating secondary cell death *in vivo*.

## RESULTS

### RNA-seq analysis identifies genes with altered expression in microglia and macrophages after acute CNS injury

After CNS injury, microglia and macrophages produce pro-inflammatory cytokines such as TNF-α, IL-1β and IL-6, which can contribute to neural cell death (Brown and Vilalta, 2015). However, evidence from *in vitro* systems suggests that they can also be neuroprotective (Bernardino et al., 2005; Carlson et al., 1999; Figiel, 2008; Jung et al., 2011; Kadhim et al., 2008; Lambertsen et al., 2009; Marchetti et al., 2004; Masuch et al., 2016; Turrin and Rivest, 2006). Furthermore, microglia and macrophages can secrete anti-inflammatory cytokines such as IL-4 and IL-10, and neurotrophic factors such as BDNF and NGF (Anwar et al., 2016; Hellewell et al., 2016; Mracsko and Veltkamp, 2014), in response to neural injury. Which of these secreted signalling molecules are neurotoxic or neuroprotective *in vivo* remains incompletely understood.

**Fig. 1.**
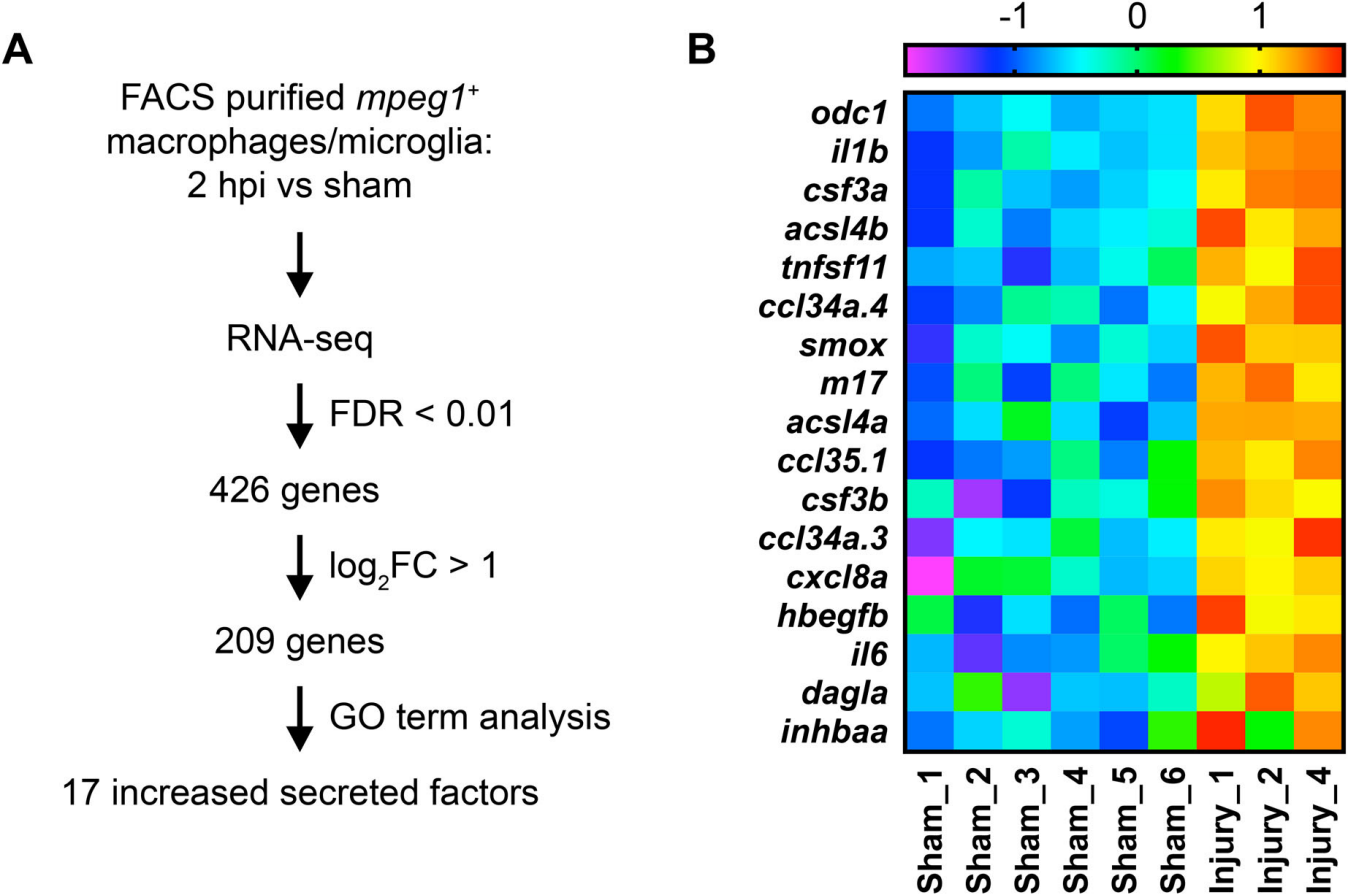
**RNA-Seq analysis reveals genes coding for secreted signalling molecules that are upregulated in microglia and macrophages after acute neural injury.** (A) Workflow for RNA-Seq gene expression profiling. FDR, false discovery rate; FC, fold change; GO, gene ontology. (B) Heat map visualising z-scores for the 17 upregulated genes coding for secreted factors identified through RNA-Seq analysis.

Like their mammalian counterparts, microglia and peripheral macrophages in larval zebrafish respond to CNS injury by migrating to the injury site, where they phagocytose neural debris (Herzog et al., 2019; Morsch et al., 2015; Ohnmacht et al., 2016; Sieger et al., 2012; Tsarouchas et al., 2018). Notably, the core microglia-specific gene expression signature is also largely conserved between zebrafish and mammals (Mazzolini et al., 2019; Oosterhof et al., 2017). To identify signalling molecules produced by microglia and macrophages after neural injury in larval zebrafish, we first assessed changes in the transcriptome of macrophage-lineage cells through RNA-seq analysis. For this, we induced acute CNS injury in *mpeg1*:GFP transgenic larvae at 4 days post fertilisation (dpf), where microglia and peripheral macrophages are labelled (Ellett et al., 2011). CNS injury was induced by piercing the optic tectum with a fine metal pin mounted on a micromanipulator. This injury paradigm induces primary and secondary cell death in neural cells of the optic tectum, peaking at 0 and 6 h post injury (hpi), respectively (Herzog et al., 2019). Both microglia and peripheral macrophages react to injury by starting to migrate towards the injury site within minutes of the insult, where they continue to accumulate in the following hours (Herzog et al., 2019; Sieger et al., 2012). We decided to isolate cephalic macrophage-lineage cells from sham and injured larvae at 2 hpi (Fig. 1A). This allowed us to study early transcriptomic changes in microglia and macrophages, which could plausibly affect the extent of secondary neural cell death at 6 hpi.

Cephalic *mpeg1*^+^ macrophage-lineage cells were isolated from larval heads by fluorescence-activated cell sorting (FACS) following an established protocol (Mazzolini et al., 2018). The gating parameters for FACS were adjusted with the help of wild-type samples so as to attain the highest possible purity of sorted *mpeg1^+^* cells (Fig. S1). We initially generated a total of 12 samples of FACS-purified macrophage-lineage cells for RNA-seq, with six samples each for the sham and 2 hpi experimental conditions. The number of *mpeg1*^+^ cells was 26,945±1176 per sample, isolated from 183±5 larvae per sample. RNA extraction from sorted cells yielded 10.6±0.8 ng of total RNA per sample. Assessment of RNA quality showed an RNA integrity number (RIN) of 10 for all samples, which represents the highest possible RIN score. The RNA thus obtained was then reverse transcribed and amplified using a commercially available kit, yielding 288±21 ng of cDNA per sample.

Libraries were prepared from cDNA and sequenced using next-generation sequencing, which generated between 23.7 and 36 million reads per sample. Reads were trimmed, mapped to the *Danio rerio* GRCz10 reference genome, counted, filtered and normalised. A principal component analysis (PCA) was then carried out on filtered and normalised expression data to explore patterns with respect to experimental groups. This revealed high duplication and low mapping rates for three samples from the 2 hpi experimental group, which did not cluster well with the other samples in PCA plots (Fig. S2). Since inclusion of these samples would have caused signals from the remaining samples to be overwhelmed, they were excluded from further analysis. Hence, all subsequent analysis was carried out using six samples for the sham experimental group, and the three remaining samples for the 2 hpi experimental group. Filtering and normalisation were repeated for these samples before proceeding. Differential analysis was then carried out to compare gene expression between the sham and 2 hpi experimental groups. This identified 426 differentially expressed genes with a false discovery rate (FDR)<0.01 (Fig. 1A). Of these, 348 were upregulated and 78 were downregulated. These results show that neural injury leads to changes in the transcriptome of macrophage-lineage cells as early as 2 hpi. Importantly, the raw and processed data from our RNA-seq analysis are available through the Gene Expression Omnibus (GEO) database (accession number GSE140810).

**Fig. 2.**
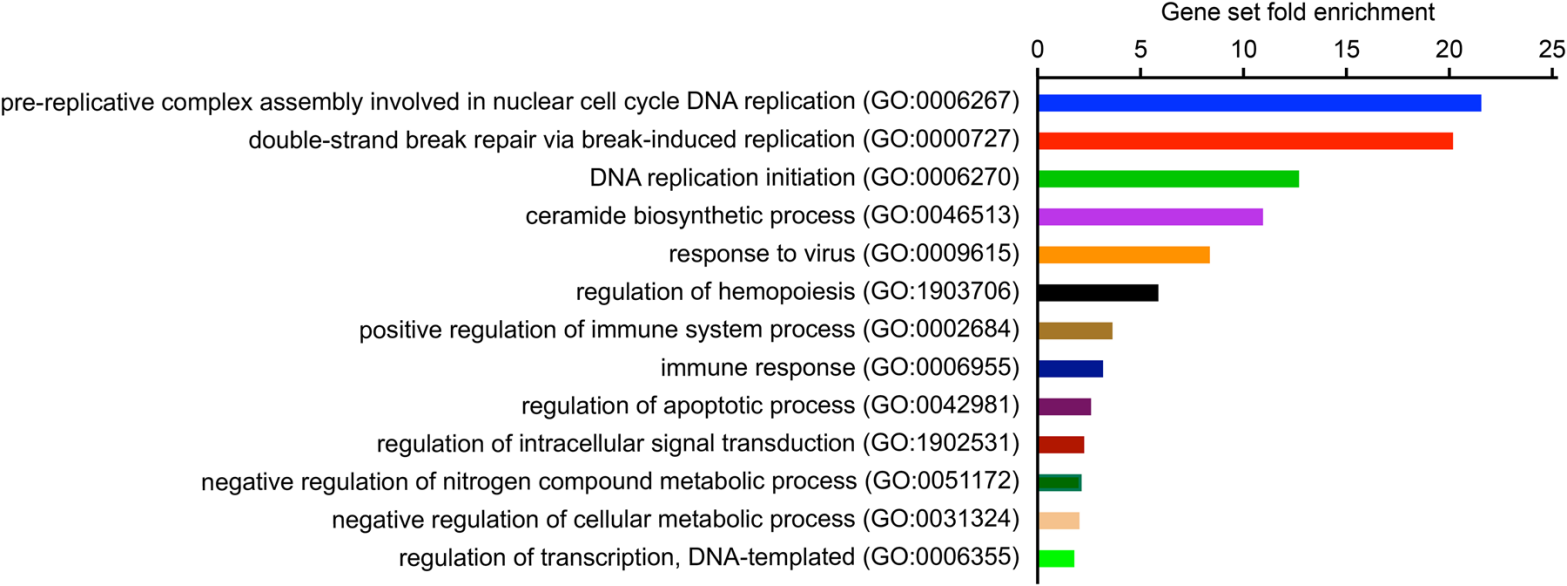
**Gene ontology analysis shows overrepresentation of genes involved in immune response, proliferation and cellular signalling and metabolism.** A PANTHER overrepresentation test was carried out to identify GO biological process categories overrepresented among the set of 426 genes with FDR<0.01.

To analyse these transcriptomic changes in more detail, we performed gene ontology (GO) analysis of our set of 426 differentially regulated genes using the PANTHER Classification system (Mi et al., 2019). More specifically, we conducted a PANTHER overrepresentation test to identify the biological processes that these differentially regulated genes are preferentially involved in (Fig. 2). Not unexpectedly, this analysis revealed an overrepresentation of immune-regulatory genes. In addition, genes involved in DNA replication were overrepresented, possibly indicating a proliferative response of macrophage-lineage cells to neural injury. Genes that regulate cellular signalling, metabolism and transcription were also overrepresented, suggesting that macrophage-lineage cells undergo profound changes in their cellular state in response to neural injury. These findings are consistent with previous research showing changes in immune regulation, proliferation and cellular metabolism in macrophage-lineage cells after CNS injury in mammals (Anwar et al., 2016; Hellewell et al., 2016; Mracsko and Veltkamp, 2014).

### Expression of a range of secreted signalling molecules is upregulated in cephalic macrophage-lineage cells after neural injury

Next, we sought to identify genes coding for secreted signalling molecules that were upregulated after neural injury, since such molecules are well placed to have a direct effect on neuronal survival. For this, we considered upregulated genes with FDR<0.01 and log_2_FC>1, of which there were 209 (Fig. 1A). Based on the GO information available for each of these genes, we identified 17 upregulated genes that encode secreted or membrane-bound signalling molecules, or enzymes that produce secreted signalling molecules, within this set (Fig. 1A). The 17 genes thus identified encode molecules from five different categories, including cytokines, chemokines, growth factors, enzymes involved in polyamine biosynthesis, and enzymes involved in arachidonic acid metabolism (Fig. 1B; Table 1). Overall, these results are in good agreement with findings from mammalian models of neural injury. In particular, microglia and macrophages are known to increase the production of cytokines, chemokines and growth factors after damage to the mammalian CNS (Anwar et al., 2016; Hellewell et al., 2016; Mracsko and Veltkamp, 2014). Likewise, the metabolism of polyamines (Kim et al., 2009; Zahedi et al., 2010) and of arachidonic acid (Adibhatla et al., 2006; López-Vales et al., 2011; Rink and Khanna, 2011) increases after neural injury in mammals, even though this has not been specifically linked to macrophage-lineage cells.

**Table 1.**
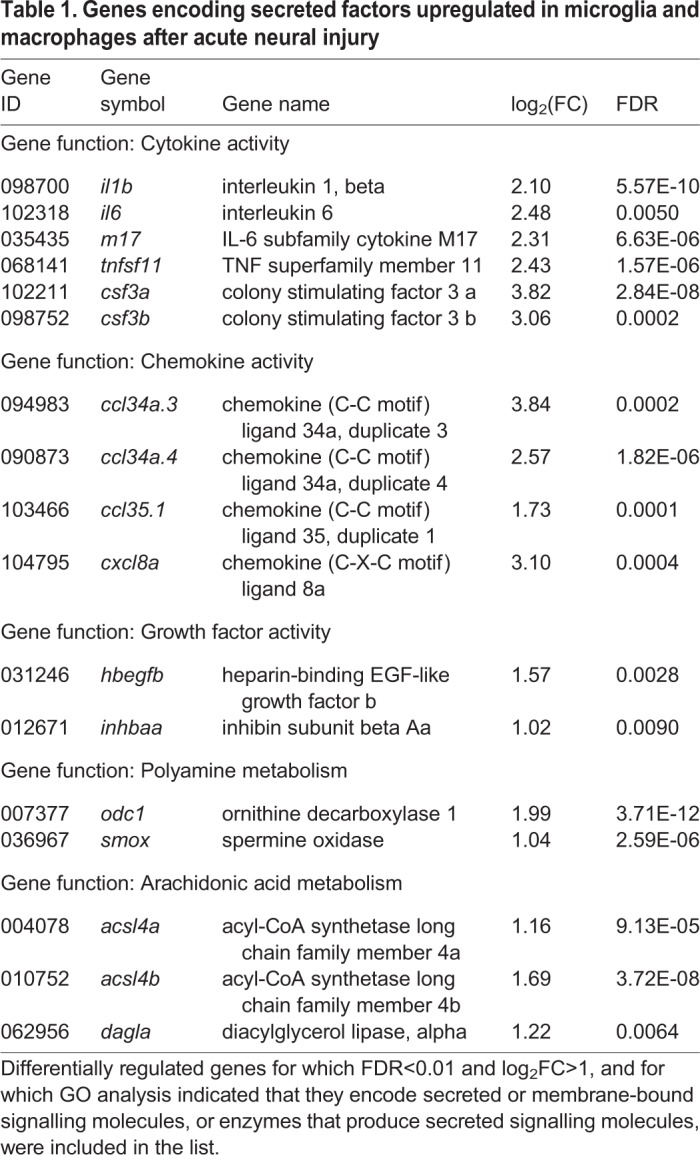
**Genes encoding secreted factors upregulated in microglia and macrophages after acute neural injury**

We hypothesised that molecules secreted by microglia and macrophages can modulate secondary cell death, and sought to test this hypothesis for a small subset of the 17 upregulated signalling molecules. For this analysis, we selected three genes from two gene categories: the cytokine *tnfsf11* and the polyamine-metabolising enzymes *odc1* and *smox*. These genes were chosen because they were among the most differentially regulated of the secreted factors (Fig. 1B, Table 1), and because we wanted to analyse the roles of different genes both within and between different gene categories.

**Fig. 3.**
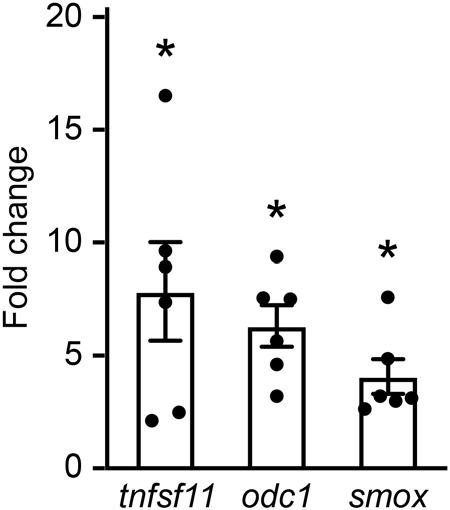
**RT-qPCR confirms changes in gene expression detected through RNA-seq analysis.** The changes in *tnfsf11, odc1* and *smox* expression in FACS-purified macrophage-lineage cells after neural injury were assessed by RT-qPCR. Each data point was generated by comparing gene expression in one cDNA sample from injured larvae at 2 hpi with that in one sample from sham larvae, with both cDNA samples generated on the same day. Three data points were derived from samples also used for RNA-seq analysis, and three from freshly generated samples. Sorted cells from about 180 larvae were pooled for each cDNA sample. Wilcoxon signed rank test was used to compare experimental groups. *P*=0.031 for sham versus injured for all three genes. *P<0.05.

To validate our RNA-seq results for *tnfsf11*, *odc1* and *smox*, we conducted RT-qPCR analysis for these three genes. We performed RT-qPCR on remaining cDNA samples from our RNA-seq experiment, and also on freshly generated cDNA samples from FACS-purified macrophage-lineage cells from sham and injured larvae at 2 hpi. These experiments indicated upregulation of all three genes after neural injury (Fig. 3). Hence, our RT-qPCR results confirmed the injury-induced increase in expression of *tnfsf11*, *odc1* and *smox* from our RNA-seq analysis.

### CRISPR/Cas9-mediated gene knockout allows for rapid assessment of the role of candidate genes in secondary cell death

We next sought to test whether *tnfsf11*, *odc1* and *smox* can modulate secondary cell death after neural injury *in vivo*. For this, we used CRISPR/Cas9-mediated gene editing, where we injected a guide RNA (gRNA) targeting a gene of interest together with Cas9 enzyme into one-cell-stage embryos. In zebrafish, this efficiently generates mutations in the targeted gene in a high proportion of cells (Jao et al., 2013). This approach allows for analysis of gene function in gRNA-injected F0 animals, which we refer to as ‘crispants’. For each of our three genes, we generated at least four gRNAs. Each gRNA’s target sequence was designed to overlap the restriction site of an endonuclease in one of the gene’s exons. This allowed us to test gRNA efficiency by assessing the extent of loss of the targeted endonuclease restriction site in gRNA-injected larvae (Fig. S3). Using this strategy, we were able to identify at least one gRNA with ≥80% efficiency for each gene (Table S1). For genes with two highly efficient gRNAs, both gRNAs were co-injected in knockout experiments.

**Fig. 4.**
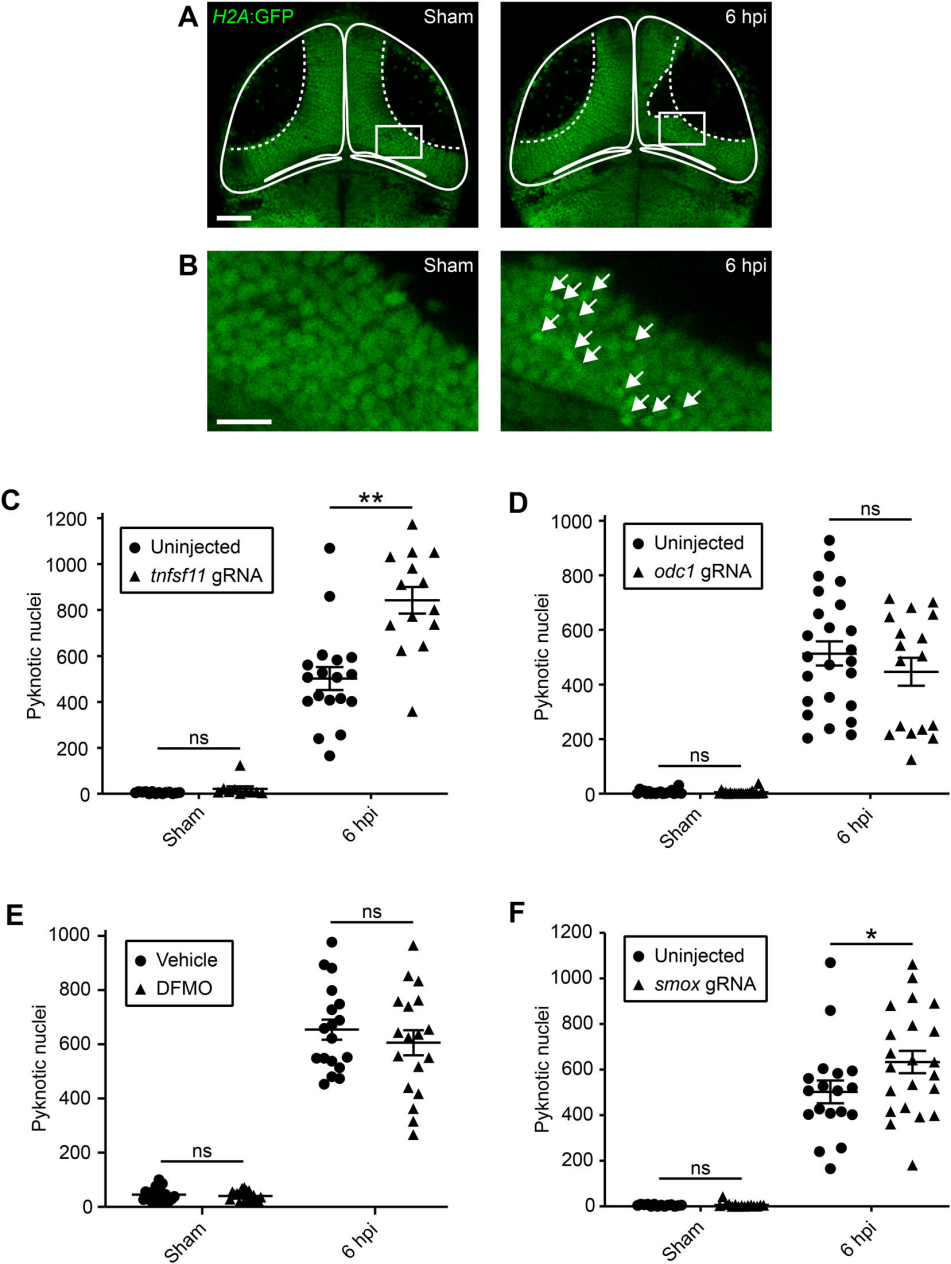
**Reducing the function of *tnfsf11* and *smox*, but not *odc1*, increases cell death after acute neural injury.** (A) Confocal images of the optic tectum of sham and injured *H2A*:GFP transgenic larvae at 6 hpi. The dashed line indicates the location of the injury site within the cell body layer. Scale bar: 50 µm. (B) Close-up of regions indicated in (A). White arrows indicate pyknotic nuclei. Scale bar: 5 µm. (C) Quantification of pyknotic nuclei within the tectum of uninjected animals, or of animals injected with *tnfsf11* gRNA. *n* (sham, uninjected)=14 animals. *n* (sham, *tnfsf11* gRNA)=10 animals. *n* (6 hpi, uninjected)=18 animals. *n* (6 hpi, *tnfsf11* gRNA)=14 animals. *N*=3 independent experiments. Two-way ANOVA with Bonferroni correction was used to compare experimental groups. *P*>0.999 for sham, uninjected versus sham, *tnfsf11* gRNA. *P*<0.001 for 6 hpi, uninjected versus 6 hpi, *tnfsf11* gRNA. **P<0.01. (D) Quantification of pyknotic nuclei within the tectum of uninjected larvae, or of larvae injected with *odc1* gRNA. *n* (sham, uninjected)=20 animals. *n* (sham, *odc1* gRNA)=21 animals. *n* (6 hpi, uninjected)=24 animals. *n* (6 hpi, *odc1* gRNA)=17 animals. *N*=4 independent experiments. Two-way ANOVA with Bonferroni correction was used to compare experimental groups. *P*>0.999 for sham, uninjected versus sham, *odc1* gRNA. *P*=0.331 for 6 hpi, uninjected versus 6 hpi, *odc1* gRNA. ns, not significant. (E) Quantification of pyknotic nuclei within the tectum of larvae treated with vehicle or DFMO. *n* (sham, vehicle)=20 animals. *n* (sham, DFMO)=18 animals. *n* (6 hpi, vehicle)=18 animals. *n* (6 hpi, DFMO)=18 animals. *N*=2 independent experiments. Two-way ANOVA with Bonferroni correction was used to compare experimental groups. *P*>0.999 for sham, vehicle versus sham, DFMO. *P*=0.494 for 6 hpi, vehicle versus 6 hpi, DFMO. (F) Quantification of pyknotic nuclei within the tectum of uninjected animals, or of animals injected with *smox* gRNA. *n* (sham, uninjected)=14 animals. *n* (sham, *smox* gRNA)=15 animals. *n* (6 hpi, uninjected)=18 animals. *n* (6 hpi, *smox* gRNA)=22 animals. *N*=3 independent experiments. Two-way ANOVA with Bonferroni correction was used to compare experimental groups. *P*>0.999 for sham, uninjected versus sham, *smox* gRNA. *P*=0.036 for 6 hpi, uninjected versus 6 hpi, *smox* gRNA. *P<0.05. Note that the data for sham and 6 hpi uninjected animals are the same in (C) and (F) since larvae in these groups were processed in the same experiment. ns, not significant.

To investigate the role of *tnfsf11*, *odc1* and *smox* in injury-induced cell death, we used our previously established experimental setup for quantification of secondary cell death (Herzog et al., 2019). For this, we induced acute neural injury in the optic tectum of *H2A*:GFP transgenic larvae (Pauls et al., 2001), where all cell nuclei are labelled (Fig. 4A). This enabled us to assess pyknosis, the condensation of chromatin in the nucleus of a dying cell, as an *in vivo* readout for cell death (Fig. 4B). Secondary cell death was quantified by manual counting of the number of pyknotic nuclei within the tectum at 6 hpi, the peak of secondary cell death in this model system (Herzog et al., 2019).

To investigate whether microinjection of gRNAs itself had an effect on the extent of secondary cell death, we first quantified cell death at 6 hpi in uninjected animals, in animals injected with Cas9 enzyme but no gRNA, and in animals injected with Cas9 enzyme and a scrambled gRNA. The scrambled gRNA is a negative control gRNA targeting the human β-globin intron mutation that causes β-thalassemia. Our results showed no difference in the levels of cell death between uninjected larvae and those injected with no gRNA or scrambled gRNA (Fig. S4). This confirmed that gRNA microinjection itself does not alter the level of secondary cell death.

### Knockout of the cytokine gene *tnfsf11* increases secondary cell death

We first investigated the role of *tnfsf11* in secondary cell death. *Tnfsf11*, also known as RANKL, TRANCE, OPGL or ODF, is a member of the tumour necrosis factor (TNF) superfamily of signalling proteins. It is a type II membrane protein that is best known as a regulator of osteoclast differentiation and immune system function (Hanada et al., 2011). *Tnfsf11* can modulate cell death through activation of the anti-apoptotic Akt/PKB signalling pathway (Wada et al., 2006), making it a plausible candidate regulator of cell death after CNS injury.

Our RNA-seq and RT-qPCR results indicate that the expression of *tnfsf11* is upregulated in macrophage-lineage cells after acute neural injury (Figs 1B and 3; Table 1). To investigate whether *tnfsf11* can regulate secondary cell death after acute neural injury, we counted the number of pyknotic nuclei at 6 hpi in *tnfsf11* crispants. If *tnfsf11* had a neurotoxic or a neuroprotective effect, we would expect to see a decrease or an increase in cell death after *tnfsf11* knockout. Interestingly, we found that CRISPR/Cas9-mediated knockout of *tnfsf11* lead to a 68% increase in cell death in injured larvae at 6 hpi (Fig. 4C). Importantly, we did not detect a difference in cell death after *tnfsf11* knockout in sham larvae (Fig. 4C), indicating that *tnfsf11* specifically modulates secondary cell death rather than changing the background level of cell death. Overall, these findings are consistent with a neuroprotective effect of *tnfsf11* in the context of acute neural injury.

### Knockout of the polyamine-metabolising gene *smox*, but not *odc1*, increases secondary cell death

Next, we sought to determine whether the polyamine-metabolising genes *odc1* and *smox* also regulate secondary cell death. Polyamines such as putrescine, spermidine and spermine are ubiquitous organic polycations whose presence effects multiple cellular processes. In the CNS, polyamines modulate the activity of a variety of ion channels including NMDA receptors (Rock and Macdonald, 1995). With NMDA receptors acting as key mediators of excitotoxicity (Dorsett et al., 2017), it is plausible that polyamines could regulate excitotoxic cell death after acute neural injury.

The *odc1* gene encodes ornithine decarboxylase, the rate-limiting enzyme in the polyamine biosynthesis pathway that catalyses the conversion of ornithine to putrescine. Interestingly, the expression of *odc1* is upregulated in macrophage-lineage cells after neural injury (Figs 1B and 3, Table 1), suggesting an increase in the rate of polyamine biosynthesis in the damaged CNS. To investigate whether this effects the extent of secondary cell death, we quantified cell death at 6 hpi in *odc1* crispants. Intriguingly, *odc1* knockout did not appear to have an effect on secondary cell death (Fig. 4D).

To confirm our result from *odc1* gene knockout, we also sought to reduce *odc1* function pharmacologically. For this, we used the ornithine decarboxylase inhibitor DFMO, which has been shown to inhibit polyamine biosynthesis in larval zebrafish (Mastracci et al., 2015; Mounce et al., 2016). Consistent with our data from *odc1* knockout, DFMO treatment had no impact on secondary cell death (Fig. 4E). Taken together, these results suggest that the overall rate of polyamine biosynthesis is not a critical factor in regulating secondary cell death.

We next sought to analyse the role of *smox* in secondary cell death. The *smox* gene encodes spermine oxidase, which catalyses the oxidation of spermine to spermidine. The expression of *smox* is upregulated in macrophage-lineage cells after neural injury (Figs 1B and 3, Table 1). Interestingly, we found that CRISPR/Cas9-mediated knockout of *smox* lead to a 26% increase in cell death in injured but not sham larvae at 6 hpi (Fig. 4F). This finding is consistent with a potential neuroprotective role of *smox* after acute neural injury. Given the role of *smox* in converting spermine to spermidine, this result also raises the possibility that the relative amounts of spermine and spermidine in the CNS may be important determinants of the extent of secondary cell death after acute CNS damage.

### An imaging-based compound screen reveals small molecule modulators of secondary cell death

To complement our work on signalling molecules secreted by macrophages and microglia, we sought to identify cellular pathways that regulate secondary cell death in a more unbiased manner. To this purpose, we conducted an imaging-based compound screen using the Enzo Life Sciences SCREEN-WELL® FDA-approved drug library, v.2.0. Each of the 786 drugs in the library is extensively annotated with regard to its mechanism of action, allowing us to infer each hit compound's target signalling pathway. Prior to the screen, we tested all compounds in the library for systemic toxicity at 5, 10 and 20 µM. In the screen, each drug was then used at its highest non-toxic concentration, which was 20 µM for the majority of the compounds.

**Fig. 5.**
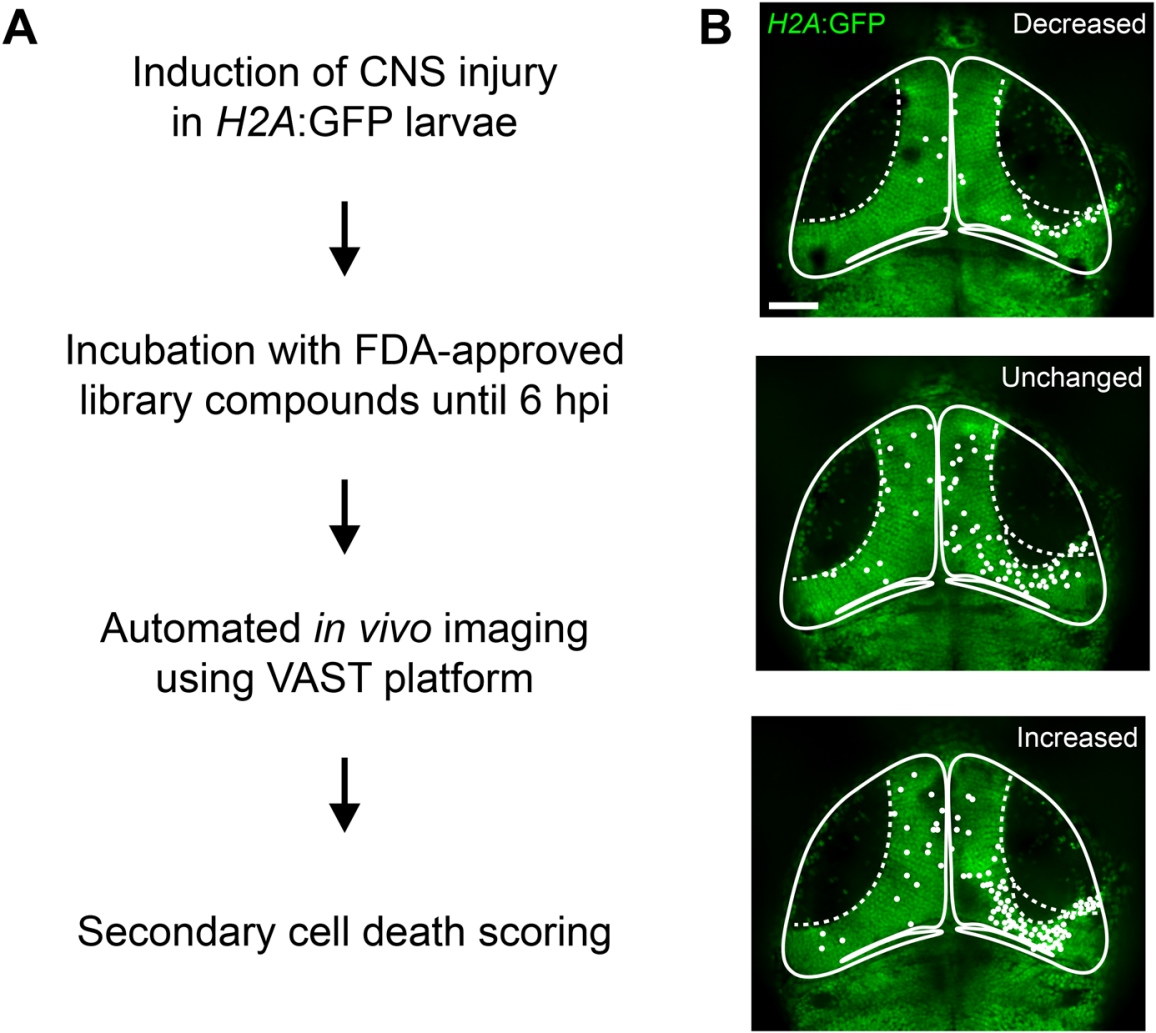
**The VAST platform enables a partially automated *in vivo* imaging-based compound screen for modulators of secondary cell death.** (A) Workflow for compound screen. A total of three rounds of screening was carried out, with putative hits from each round being reassessed in the next round. (B) VAST images of the optic tectum of *H2A*:GFP transgenic larvae at 6 hpi, illustrating the three cell-death scoring categories used in the compound screen. The extent of cell death in drug-treated larvae was scored relative to that in DMSO-treated control larvae by visual assessment of the amount of pyknotic nuclei. The dashed line indicates the location of the injury site within the cell body layer. White circles indicate pyknotic nuclei. Scale bar: 50 µm.

For the compound screen, we adapted and partially automated our *in vivo* cell-death assay (Fig. 5A). Neural injury was induced in the optic tectum of *H2A*:GFP transgenic larvae at 4 dpf. Larvae were then transferred into 96-well plates, and compounds from the library were added to each well. At 6 hpi, larvae were automatically imaged using a Vertebrate Automated Screening Technology (VAST) system. The VAST platform combines a large particle sampler and a VAST BioImager (Pardo-Martin et al., 2010) with a customised spinning disk confocal microscope, thus enabling automated mounting and imaging of zebrafish larvae. This setup has previously been used to identify compounds that regulate myelination in larval zebrafish (Early et al., 2018), highlighting its suitability for mechanistic investigations of cellular processes in the larval zebrafish CNS.

To identify compounds that modulate secondary cell death, we then analysed the images acquired by the VAST platform. To expedite the image-analysis process, we used a scoring system where the extent of cell death in drug-treated larvae was categorised relative to that in DMSO-treated larvae through visual inspection of the number of pyknotic nuclei in z-stacks of images, rather than through time-consuming manual counting of pyknotic nuclei. The scoring categories were ‘decreased’, ‘unchanged’ or ‘increased’ cell death (Fig. 5B). Compounds that appeared to cause nervous system toxicity as shown by excessive cell death in the optic tectum were excluded from further analysis. To assess the reliability of this scoring system, we conducted an experiment with a small number of compounds for which scoring had indicated a change in the extent of cell death during the initial phase of the screen. In this experiment, we assessed the extent of cell death through scoring and also quantified the number of pyknotic nuclei at 6 hpi through manual counting in the same larvae. The scoring results were confirmed through counting for two of every four compounds (Table S2), indicating that the scoring system can detect compounds that modulate cell death. Importantly, previous small molecule screens for modulators of neural regeneration in larval zebrafish have successfully used similar scoring strategies (Bremer et al., 2017; Namdaran et al., 2012).

In the initial screen of all 786 compounds from the library, we identified 22 putative hit compounds. Of these, six decreased cell death, whereas 16 increased it (Table 2). We then conducted two further rounds of screening to reassess these initial results. In the first re-screen, we re-tested the 22 putative hit compounds using remaining drugs from the library. This confirmed our results from the initial screen for ten putative hit compounds, of which two decreased and eight increased cell death (Table 2). In the second re-screen, we re-tested the ten putative hit compounds from the first re-screen using freshly ordered compounds. We also included sham larvae, which enabled us to exclude compounds that change the background level of cell death instead of specifically modulating secondary cell death. The second re-screen yielded two putative hit compounds that specifically regulate secondary cell death, with one compound decreasing and one increasing it (Table 2).

**Table 2.**
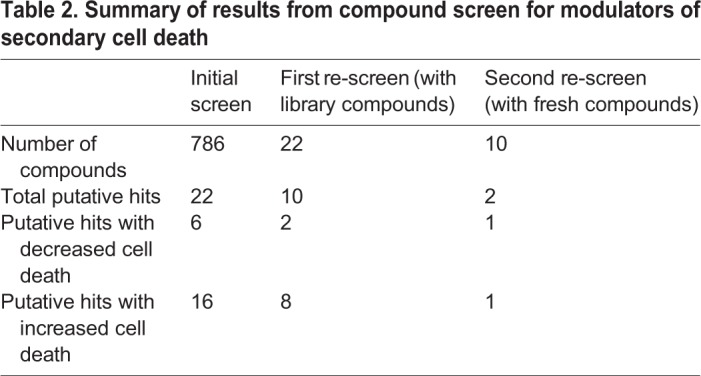
**Summary of results from compound screen for modulators of secondary cell death**

**Fig. 6.**
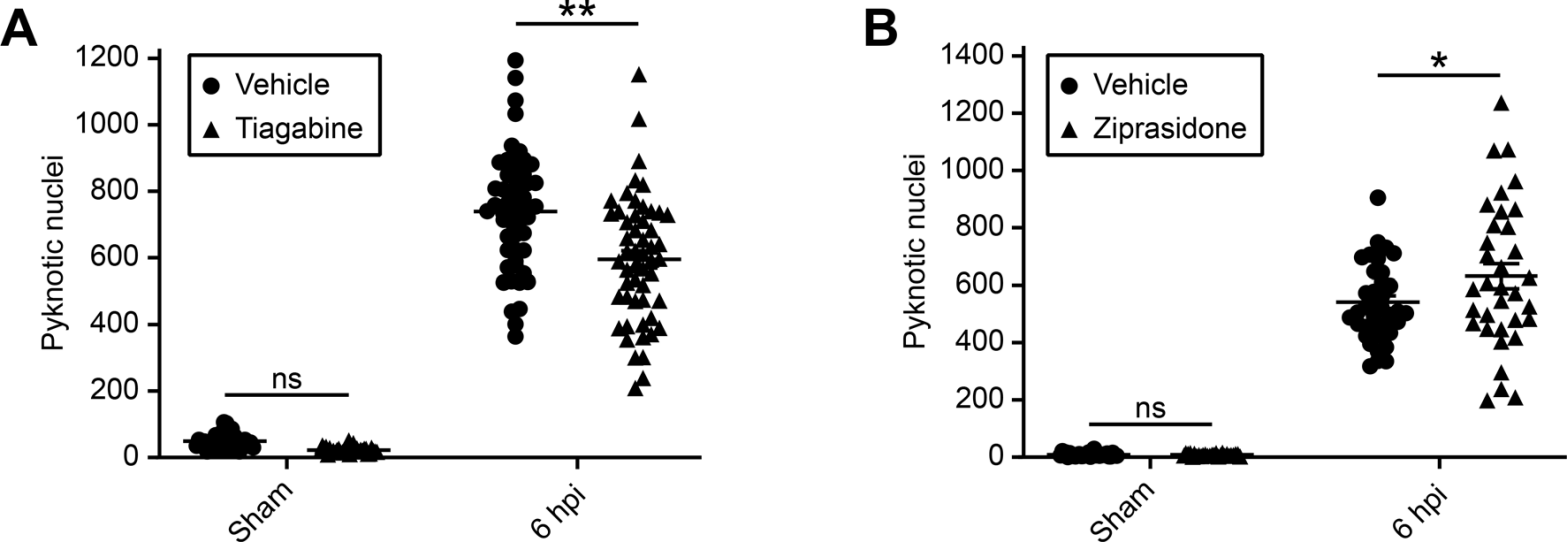
**The hit compounds tiagabine and ziprasidone change the level of secondary cell death.** (A) Quantification of pyknotic nuclei within the tectum of larvae treated with vehicle or tiagabine. *n* (sham, vehicle)=32 animals. *n* (sham, tiagabine)=33 animals. *n* (6 hpi, vehicle)=53 animals. *n* (6 hpi, tiagabine)=53 animals. *N*=2 independent experiments. Two-way ANOVA with Bonferroni correction was used to compare experimental groups. *P*=0.936 for sham, vehicle versus sham, tiagabine. *P*<0.001 for 6 hpi, vehicle versus 6 hpi, tiagabine. ns, not significant. **P<0.01. (B) Quantification of pyknotic nuclei within the tectum of larvae treated with vehicle or ziprasidone. *n* (sham, vehicle)=31 animals. *n* (sham, ziprasidone)=27 animals. *n* (6 hpi, vehicle)=41 animals. *n* (6 hpi, ziprasidone)=34 animals. *N*=2 independent experiments. Two-way ANOVA with Bonferroni correction was used to compare experimental groups. *P*>0.999 for sham, vehicle versus sham, ziprasidone. *P*=0.024 for 6 hpi, vehicle versus 6 hpi, ziprasidone. ns, not significant. *P<0.05.

The putative hit compounds that decreased and increased cell death were the antiepileptic drug tiagabine and the antipsychotic medication ziprasidone, respectively. To further confirm the effect of these putative hits on secondary cell death, we then quantified the number of pyknotic nuclei at 6 hpi through manual counting, in two additional experiments for each compound. Importantly, this substantiated our results from the compound screen for both drugs. More specifically, we found a 19% decrease in secondary cell death for tiagabine (Fig. 6A) and a 17% increase for ziprasidone (Fig. 6B). The extent of cell death in sham larvae was not affected by either compound (Fig. 6A,B).

Hence, our drug screen yielded two hit compounds that consistently and specifically modulate secondary cell death after acute neural injury *in vivo*.

## DISCUSSION

In the present study, we used our previously established experimental setup for quantification of secondary cell death in larval zebrafish (Herzog et al., 2019) to identify signalling pathways that modulate injury-induced cell death after acute neural injury *in vivo*. To this purpose, we combined this platform with RNA-seq gene expression profiling and compound screening.

We used RNA-seq analysis to investigate early changes in the transcriptome of cephalic macrophage-lineage cells in larval zebrafish after acute CNS injury. Our analysis revealed rapid and pronounced transcriptomic changes in these cells, with more than 400 differentially expressed genes at 2 hpi. Genes associated with the immune response, DNA replication, and cellular signalling and metabolism are overrepresented among the differentially expressed genes (Fig. 2). These results are in agreement with other transcriptomic studies from both mammals and zebrafish. RNA-seq analysis of microglia after spinal cord injury in mice revealed upregulation of genes associated with the immune response and cellular proliferation (Noristani et al., 2017). Likewise, infiltrating macrophages increase the expression of immune-system and metabolic genes after spinal cord injury in mice (Zhu et al., 2017). In addition, the expression of genes involved in cell cycle control and DNA replication was shown to change in microglia after acute neuronal ablation in the brain of adult zebrafish (Oosterhof et al., 2017). Conversely, another study in adult zebrafish found that genes upregulated in retinal microglia after neuronal ablation are mostly associated with vesicle trafficking (Mitchell et al., 2019). This discrepancy might be due to the later time point of analysis in the latter study, capturing microglia in a regenerative state rather than during the acute phase of the injury. Overall, the transcriptional response of macrophage-lineage cells to acute neural injury appears to be conserved across vertebrate species, highlighting the relevance of our model system for the study of microglia and macrophage reactions to CNS injury. Importantly, all raw and processed RNA-seq data from our analysis are available through the GEO database (accession number GSE140810). This dataset will provide a powerful new resource for studies aiming to understand early immune-cell reactions to CNS injury.

Which aspects of the response of microglia and macrophages to CNS injury have neurotoxic or neuroprotective effects is the subject of ongoing debate (Hu et al., 2015; Li and Barres, 2018). Here we decided to focus on the role of secreted signalling molecules, since these have the potential to directly influence neuronal survival. Our RNA-seq analysis revealed 17 upregulated genes encoding such factors, including cytokines, chemokines, growth factors and enzymes involved in polyamine and arachidonic acid biosynthesis (Table 1). These results highlight the complexity of the transcriptional response of macrophage-lineage cells to neural injury.

One of the cytokines that we found to be upregulated after neural injury is the TNF superfamily member *tnfsf11* (Fig. 1B, Table 1). Similarly, DNA microarray analysis revealed *tnfsf11* upregulation in rat brain tissue after focal ischemia (Schmidt-Kastner et al., 2002). It is also upregulated after brain ischemia in mice, where its expression is restricted to microglia and macrophages (Shimamura et al., 2014). In addition, another study found an increase in expression after mechanical brain injury in mice (Kelso et al., 2015). Interestingly, we found that *tnfsf11* knockout is associated with an increase in secondary cell death (Fig. 4C), suggestive of a potential neuroprotective role. Likewise, infarct volume after ischemia in mice is increased after treatment with anti-*tnfsf11* antibody but reduced in the presence of recombinant *tnfsf11* (Shimamura et al., 2014). Furthermore, a peptide designed to specifically enhance *tnfsf11* signalling in microglia and macrophages decreases infarct volume after ischemia in mice (Kurinami et al., 2016; Shimamura et al., 2018). Together with our results, these findings indicate that *tnfsf11* is upregulated after CNS injury and provide evidence for a neuroprotective role of *tnfsf11* that is conserved across vertebrates.

We found that expression of *odc1* and *smox*, two genes involved in polyamine metabolism, is also upregulated in macrophage-lineage cells after neural injury (Fig. 1B, Table 1). *Odc1* is the rate-limiting enzyme in the polyamine biosynthesis pathway, whose expression level controls the overall rate of polyamine metabolism. Consistent with our results, *odc1* mRNA expression is upregulated after cerebral ischemia in gerbils (Kindy et al., 1994), and its enzymatic activity is increased after spinal cord injury (Mautes et al., 1999), mechanical brain injury (Henley et al., 1996; Rao et al., 1998) and cerebral ischemia (Babu et al., 2003) in rats. Intriguingly, we did not detect an effect of *odc1* gene knockout (Fig. 4D) or inhibition of *odc1* enzymatic activity with DFMO (Fig. 4E) on secondary cell death, arguing against a major role of *odc1* in this process. Findings on the role of *odc1* in cell death after CNS injury in mammals are conflicting. DFMO treatment decreases delayed neuronal cell death after cerebral ischemia in gerbils (Kindy et al., 1994). Conversely, different levels of putrescine in the mouse brain are not associated with differences in neuronal death after ischemia (Lukkarainen et al., 1995). Furthermore, administration of exogenous putrescine reduces delayed cell death after ischemia in gerbils (Gilad and Gilad, 1991), and treatment with DFMO (Lukkarinen et al., 1999) or *odc1* antisense oligonucleotides (Rao et al., 2001) increases infarct volume after ischemia in rats. Overall, the role of *odc1* in cell death after neural injury will require further investigation.

The *smox* gene, whose product converts spermine to spermidine, is also upregulated in macrophage-lineage cells after neural injury (Fig. 1B, Table 1). Interestingly, increased expression of rat *smox* mRNA occurs in the hours after ischemia (Fan et al., 2019) but not mechanical brain injury (Zahedi et al., 2010). Furthermore, the levels of spermine and spermidine in brain tissue are decreased after ischemia (Adibhatla et al., 2002) but unchanged after mechanical brain injury (Henley et al., 1996; Zahedi et al., 2010) in rats. Our results further indicated a potential neuroprotective role for *smox*, since *smox* gene knockout leads to increased neuronal cell death after CNS injury (Fig. 4F). Conversely, inhibition of *smox* enzymatic activity reduced injury volume after mechanical brain injury (Doğan et al., 1999) or ischemia (Adibhatla et al., 2002) in rats. Likewise, ischemic neuronal cell death in rats was decreased after *smox* downregulation through RNA interference (Fan et al., 2019). Consistent with these findings, treatment with exogenous spermine reduced delayed cell death after ischemia in gerbils (Gilad and Gilad, 1991) and decreased injury volume following ischemia in rats (Coert et al., 2000). However, spermine was found to exacerbate ischemic neuronal injury in mice (Duan et al., 2011). Hence, it is likely that additional research will be required to define the role of *smox* in neuronal damage after acute CNS injury.

Our CRISPR/Cas9-mediated knockout of *tnfsf11* and *smox* indicated potential neuroprotective roles for both genes. Of note, our experimental approach did not allow for gene knockout specifically in macrophage-lineage cells. Thus, knockout of *tnfsf11* and *smox* in cells other than microglia and macrophages may have contributed to the increase in secondary cell death in *tnfsf11* and *smox* crispants. Nevertheless, our results highlight the suitability of our experimental strategy for rapid identification of neuroprotective factors after acute neural injury *in vivo*.

In addition to our RNA-seq analysis of macrophage-lineage cells, we also performed an imaging-based compound screen to identify modulators of secondary cell death in a more unbiased manner. While our previous work had assessed the effect of a small number of drugs on secondary cell death (Herzog et al., 2019), a systematic survey of a larger number of molecules has not been carried out. For the screen, we used the Enzo Life Sciences SCREEN-WELL® FDA-approved drug library, v.2.0. Being FDA-approved and in use in the clinic, all compounds in the library have well-characterised bioactivity, safety and bioavailability profiles, which might help to accelerate drug development from hit compounds.

We carried out a total of three rounds of screening, with one initial screen and two re-screens of putative hit compounds from the previous rounds (Table 2). This strategy allowed us to repeatedly assess the effect on cell death seen with a particular compound, and thereby eliminate any false positives. It is possible that some of the drugs that did not affect cell death when tested at 20 µM in the initial screen would have had an effect at a higher concentration, and therefore constitute false negatives. Furthermore, we did not investigate whether drugs that showed nervous system toxicity in the initial screen would have specifically affected secondary cell death at a lower concentration. Hence, we cannot exclude the possibility that some of these drugs also represent false negatives. Overall, our screen of 786 small molecules yielded two compounds that consistently and specifically modulate secondary cell death, constituting a 0.3% hit rate. Two previous studies describing small molecule screens for modulators of neural regeneration in larval zebrafish reported hit rates of 0.5% (Namdaran et al., 2012) and 4.4% (Bremer et al., 2017). This indicates that while relatively low, our hit rate is still within the range that could reasonably be expected in this type of screen.

Of our two hit compounds, one decreases and one increases injury-induced cell death (Table 2). The hit compound that reduces secondary cell death was tiagabine (Fig. 6A), which is used in the treatment of epilepsy. Tiagabine increases the level of GABA in the CNS by blocking the neuronal GABA transporter 1, and it is hence classified as a GABA reuptake inhibitor (Meldrum and Chapman, 1999). In contrast to tiagabine, the antipsychotic medication ziprasidone, an antagonist of serotonin and dopamine receptors (Seeger et al., 1995), increases secondary cell death (Fig. 6B). The identity of our hit compounds suggests that cellular signalling through the neurotransmitters GABA, serotonin and dopamine may play important roles in regulating injury-induced cell death after acute CNS damage.

Consistent with our finding of reduced secondary cell death in the presence of tiagabine (Fig. 6A), tiagabine displays neuroprotective properties in mammalian models of acute CNS injury, potentially by reducing glutamate-mediated excitotoxicity (Green et al., 2000). More specifically, tiagabine reduces glutamate levels after ischemia in gerbils, and this is associated with decreased neuronal cell death (Inglefield et al., 1995; Iqbal et al., 2002). This result was confirmed in rats, where tiagabine treatment reduces cell death (Johansen and Diemer, 1991) as well as injury volume (Chen Xu et al., 2000; Yang et al., 2000) after ischemia. Our results further support a neuroprotective effect for tiagabine after acute neural injury.

Unlike tiagabine, ziprasidone increased secondary cell death (Fig. 6B). Interestingly, this contradicts studies in rats, which showed a reduction in infarct volume after ischemia in the presence of ziprasidone (Kaengkan et al., 2013; Kam et al., 2012). The reasons for this discrepancy remain unclear, but might include differences in timing or dosage of drug treatment. Hence, more research will be needed to understand the effect of ziprasidone on cell death after acute neural injury.

Overall, the work presented here suggests roles for multiple signalling pathways in the regulation of secondary cell death *in vivo*, including the TNF, polyamine, GABA, serotonin- and dopamine-signalling systems. This highlights the complexity of the cellular mechanisms that underlie secondary cell death. Our study did not address whether these signalling systems interact to regulate cell death after neural injury *in vivo*. Hence, the extent to which they are diverse and independent will be an interesting subject for further investigation.

Importantly, our study provides evidence for a neuroprotective role of the gene *tnfsf11* and the small molecule tiagabine, thereby confirming and extending findings from mammals. This strengthens the case for using *tnfsf11* and tiagabine as starting points for the development of novel neuroprotective treatments for patients with CNS injury.

## MATERIALS AND METHODS

### Fish husbandry

Zebrafish (*Danio rerio*) of both sexes were used in this study. All animals were maintained under standard conditions (Westerfield, 2007), and experiments were performed in accordance with British Home Office regulations. Wild-type animals were of the AB or WIK strains. Transgenic lines were *H2A.F/Z*:GFP (Pauls et al., 2001), referred to as *H2A*:GFP, and *mpeg1*:GFP (Ellett et al., 2011). If necessary, larvae were treated with 100 µM N-phenylthiourea (PTU) to inhibit melanogenesis. All chemicals were supplied by Sigma-Aldrich unless otherwise stated.

### Induction of acute CNS injury

Neural injury in larval zebrafish was induced as previously described (Herzog et al., 2019). Briefly, zebrafish larvae at 4 dpf were anaesthetised using 0.01% Ethyl 3-aminobenzoate methanesulfonate (MS-222), and mounted in 1% low-melting-point agarose (Life Technologies) with their dorsal side facing upward. The optic tectum was then injured under visual guidance of a stereomicroscope using a fine metal pin (Fine Science Tools) mounted on a micromanipulator (Narishige International Ltd.). For induction of injury, the tip of the metal pin was inserted into the optic tectum to a depth of approximately 200 µm, and subsequently retracted. After injury, larvae were carefully released from the agarose and allowed to recover for varying amounts of time depending on experimental requirements. Sham animals were anaesthetised, embedded in agarose and subsequently released as described, but not injured.

### FACS purification and RNA extraction of cephalic macrophage-lineage cells

To obtain RNA from macrophage-lineage cells for RNA-seq analysis and RT-qPCR, GFP^+^ cells were isolated from *mpeg1*:GFP larvae using FACS, and RNA was extracted from sorted cells as previously described (Mazzolini et al., 2018). All steps were performed at 4°C to preserve RNA integrity. About 180 larvae were used to generate each sample. Briefly, sham or injured larvae at 2 hpi were anaesthetised and larval heads were transected using surgical microscissors (Fine Science Tools). Heads were then transferred into a glass homogeniser containing 1 ml homogenisation medium (15 mM HEPES, 2 mM D-Glucose in HBSS) and a cell suspension was generated by homogenising the tissue. The cell suspension was run through a 40 µm cell strainer, after which cells were pelleted by centrifugation. The cell pellet was then resuspended in 1 ml 22% Percoll and overlaid with 0.5 ml DPBS to create a density gradient. After centrifugation, the supernatant consisting of DPBS, myelin-containing interphase and Percoll was discarded and the cell pellet was washed with 0.5 ml homogenisation medium with 2% NGS. The pellet was then resuspended in 0.5 ml homogenisation medium with 2% NGS and run through a 35 µm cell-strainer cap into 5 ml FACS tubes. GFP^+^ cells were isolated from the cell suspension using a FACSAria II flow cytometer. The sorting gate for GFP^+^ cells from *mpeg1*:GFP larvae was calibrated using wild-type larvae undergoing the same protocol in order to attain the highest possible purity of sorted GFP^+^ cells (Fig. S1). Cells were sorted directly into 1.5 ml tubes containing 1 ml of RNAProtect Cell Reagent (Qiagen) to stabilise RNA and stored at 4°C for up to 3 days before RNA extraction. Sorted cells were then pelleted and RNA was extracted from the pellet using an RNeasy Plus Micro kit (Qiagen). RNA quantity and quality were assessed on a LabChip^®^ GX Touch™ 24 nucleic acid analyser (PerkinElmer).

### RNA-seq library preparation, sequencing and analysis

To generate cDNA samples for RNA-seq gene expression profiling, RNA was reverse transcribed and amplified using the Ovation^®^ RNA-Seq System V2 (NuGEN). Amplified cDNA was purified using a MinElute Reaction Cleanup Kit (Qiagen) and quantified on a NanoDrop One spectrophotometer (Thermo Fisher Scientific).

Library preparation, sequencing and bioinformatics analysis were carried out by Edinburgh Genomics. Libraries were prepared for each sample using a manual TruSeq DNA Nano gel-free library kit (Illumina). The samples were then sequenced on a NovaSeq 6000 instrument. Sequencing reads were trimmed in Cutadapt (Martin, 2011) and aligned to the *Danio rerio* GRCz10 reference genome in STAR (Dobin et al., 2013). Reads were assigned to features of type ‘exon’ using featureCounts (Liao et al., 2014). The raw-counts table thus generated was filtered to remove genes consisting predominantly of near-zero counts and reads were normalised using the weighted trimmed mean of M-values method (Robinson and Oshlack, 2010). A principal components analysis was then undertaken on filtered and normalised expression data to explore patterns with respect to experimental factors. The cumulative proportion of variance associated with each factor was used to study the level of structure in the data, while associations between continuous value ranges in principal components and categorical experimental factors was assessed with an ANOVA test. Differential analysis was carried out with edgeR (Robinson et al., 2010) to compare gene expression between sham and 2 hpi experimental groups. Fold changes were estimated as per the default behaviour of edgeR, to avoid artefacts which occur with empirical calculation. Statistical assessment of differential expression was carried out using a quasi-likelihood *F*-test.

### GO overrepresentation analysis

GO overrepresentation analysis was carried out using an online tool (http://www.pantherdb.org) based on the PANTHER classification system (Mi et al., 2019). For this, a list of differentially expressed genes with FDR<0.01 was uploaded alongside a list of all genes detected in our RNA-seq experiment. A statistical overrepresentation test was then conducted using the ‘GO Biological process complete’ annotation set.

### Generation of expression heatmap

To generate an expression heatmap for the differentially regulated genes that encode secreted factors, z-scores for every sample were calculated from normalised counts for each gene. Heatmaps were created by plotting z-scores in Prism (GraphPad).

### RT-qPCR

For RT-qPCR, cDNA was synthesised from RNA using the iScript™ cDNA synthesis kit (Bio-Rad). RT-qPCR was performed on a LightCycler 96 Real-Time PCR system using SsoAdvanced™ Universal SYBR Green Supermix (Bio-Rad). Primer efficiencies were determined from cDNA dilution curves. The sequences, product sizes, optimal concentrations and efficiencies of primers used for RT-qPCR are listed in Table S3. Analysis of RT-qPCR data was carried out using the Pfaffl Method, which takes primer efficiency into account (Pfaffl, 2001).

### CRISPR/Cas9-mediated gene editing

gRNAs were designed manually to target endonuclease restriction sites in exonic sequences of genes to be knocked out. At least four gRNAs were generated for each gene, and their efficiencies were tested by assessing the extent of loss of their targeted restriction sites through restriction fragment length polymorphism analysis. For this, 1 nl injection solution containing crRNAs and tracrRNA (all at 250 ng/µl) together with Cas9 enzyme (NEB) was injected into one-cell-stage embryos. PCR was performed on genomic DNA extracted from individual larvae at 24 hpf. The sequences, target exons and product sizes of primers used for gRNA efficiency testing are listed in Table S4. PCR products were digested using the appropriate restriction enzyme (Table S1), and restriction fragments were separated through agarose gel electrophoresis. The efficiency of each gRNA was assessed by visual inspection of the relative brightness of the different restriction fragments (Fig. S3). For this, gel images were carefully inspected by at least two independent experienced investigators for each gRNA. This method for assessing gRNA efficiency has previously been validated through direct sequencing of gRNA target sites (Herzog et al., 2019; Tsarouchas et al., 2018). Only gRNAs with ≥80% efficiency were used in knockout experiments. The target sequences, target exons, restriction enzymes and efficiencies of these gRNAs are listed in Table S1. The target sequence of the scrambled gRNA was 5′-CCTCTTACCTCAGTTACAATTTATA-3′, which comprises the human β-globin intron mutation that causes β-thalassemia.

### Drug treatments

Pharmacological agents were delivered by bath application. The following drugs were used: 10 mM DFMO (Tocris Bioscience); 20 µM imiquimod; 20 µM lindane; 20 µM tiagabine; 20 µM levothyroxine; and 20 µM ziprasidone (Fluorochem) in 1% DMSO. For vehicle treatment, 1% DMSO without drug was added to the embryo medium. Drugs were added to the embryo medium immediately after neural injury.

### Image acquisition and analysis

For confocal imaging of crispants, zebrafish larvae were anaesthetised and mounted in low-melting-point agarose with their dorsal side facing upward. The agarose was then covered with embryo medium to prevent desiccation during imaging. Confocal imaging was conducted on a Zeiss LSM 880 laser scanning microscope. For z-stacks of the optic tectum, images with 3.6 µm intervals between optical planes were acquired to a depth of 110 µm, starting at the dorsal side of the tectum.

Quantification of cell death was carried out as previously described (Herzog et al., 2019). Briefly, pyknotic nuclei within the tectum of *H2A*:GFP animals were manually counted in ImageJ (https://imagej.nih.gov/ij) across both tectal hemispheres and across all consecutive images of z-stacks of the optic tectum. Pyknotic nuclei display decreased size and increased brightness when compared to live nuclei (Herzog et al., 2019), and these parameters were used as the main criteria for identifying individual nuclei as pyknotic.

### Small molecule screen

We used the Enzo Life Sciences SCREEN-WELL® FDA-approved drug library, v.2.0, which contains 786 compounds of known bioactivity, safety and bioavailability. Library compounds are predominantly supplied at 10 mM in DMSO, with a small number of compounds supplied at different concentrations or in water.

Before the start of the screen, all compounds were tested for systemic toxicity at a range of concentrations. For this, wild-type larvae at 4 dpf were distributed into 96-well plates at a density of three larvae per well, and incubated with library compounds at 5, 10 or 20 µM in 300 µl embryo medium with PTU and 1% DMSO for 6 h. Larvae were then assessed for indicators of systemic toxicity such as lethality or abnormal body shape. In the screen, each compound was used at the highest concentration that did not induce systemic toxicity. Compounds that showed systemic toxicity at 5 µM were used at 1 µM in the screen.

In the initial screen, acute neural injury was induced in *H2A*:GFP larvae, and larvae were transferred into 96-well plates at a density of two larvae per well. For each library compound, six larvae were incubated with the compound in 300 µl embryo medium with PTU and 1% DMSO for 6 h. Larvae were then anaesthetised and automatically imaged using the VAST system (Pardo-Martin et al., 2010). This platform uses a Large Particle Sampler (Union Biometrica) to transfer larvae from each well of a 96-well plate to a BioImager (Union Biometrica), where it automatically orients them within a thin-walled glass capillary to allow imaging from the dorsal side. The BioImager platform is combined with a Zeiss Axio Examiner D1 microscope, fitted with a high-speed CSU-X1 spinning disk confocal scanner and a Zeiss AxioCam 506m CCD camera. For z-stacks of the optic tectum, 20 images with 10 µm intervals between optical planes were acquired. Larvae were imaged in the same order as compounds were added to the plate in order to ensure similar treatment times across larvae.

Image files from the VAST system were processed to attach well-position information (Early et al., 2018). The extent of cell death in drug-treated larvae relative to that in DMSO-treated larvae was then manually scored in ImageJ. Scoring was conducted by visual inspection of the amount of pyknotic nuclei in z-stacks of images, with images from DMSO-treated and drug-treated larvae being viewed side by side. Scoring categories were ‘decreased’, ‘unchanged’ or ‘increased’ cell death (Fig. 5B). Drug-treated larvae from each plate were scored relative to DMSO-treated larvae from the same plate in order to control for variation between experiments. All scoring was performed by the same investigator.

In the first re-screen, putative hits from the initial screen were re-tested with remaining compound from the library using the same experimental parameters as in the initial screen. In the second re-screen, putative hits from the first re-screen were re-tested with freshly ordered compound. Both sham and injured larvae were included in the second re-screen, and 12 larvae per experimental group were tested.

To quantify cell death in larvae treated with DFMO, imiquimod, lindane, tiagabine, levothyroxine or ziprasidone, z-stacks of the optic tectum were acquired on the VAST system. Pyknotic nuclei within the optic tectum were manually counted in ImageJ across both tectal hemispheres and across 13 consecutive optical planes from the z-stack, starting at the dorsal side of the tectum.

### Experimental design and statistical analysis

Animals were randomly allocated to experimental groups before the start of each experiment. Researchers were blinded to experimental group for data analysis.

All population data are presented as mean±SEM. Statistical analysis was performed using Prism. Briefly, data sets were assessed for normality, and appropriate statistical tests were carried out as stated in the figure legends.

## Supplementary Material

Supplementary information
