# Supplementary material for: RNA-seq analysis and compound screening highlight multiple signalling pathways regulating secondary cell death after acute CNS injury in vivo
Source: Biol Open. 2020 May 4;9(5):bio050260. doi: 10.1242/bio.050260 (PMC7225090; doi:10.1242/bio.050260)
Supplement: Supplementary information [file biolopen-9-050260-s1.pdf]

Figure S1

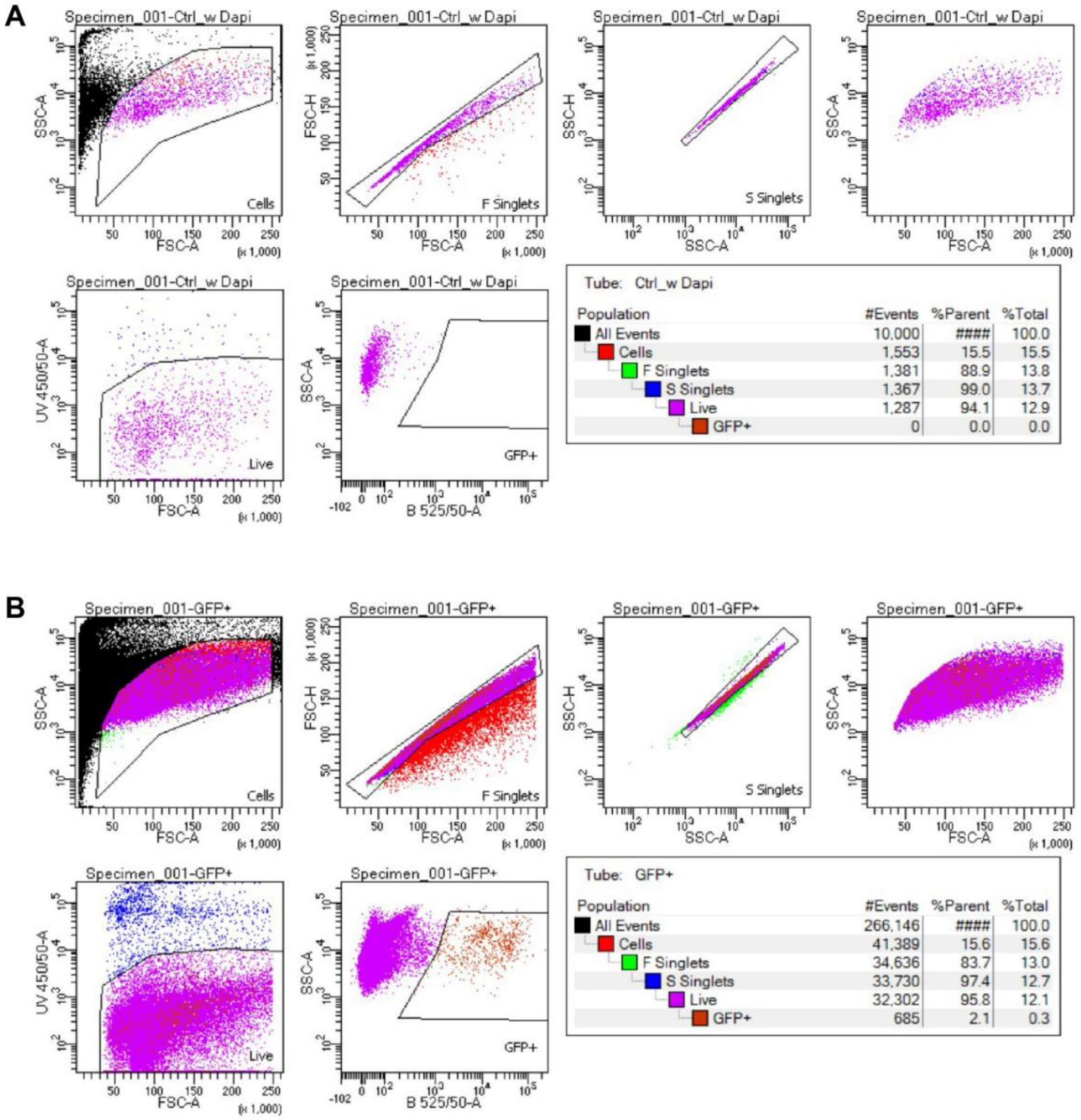

Fig. S1. Flow cytometry was used to isolate GFP$^+$ cells from *mpeg1:GFP* larvae. Representative FACS plots and population reports for sorting of cell suspensions from wild type (A) and *mpeg1:GFP* (B) larvae. Successive gating allowed for the sequential selection first of cells by forward and sideward scatter, then single cells by forward and sideward scatter, then live cells by exclusion of DAPI staining, and then GFP$^+$ cells by green fluorescence. The wild type sample was used to adjust gating parameters such that autofluorescent cells were excluded from the population of sorted GFP$^+$ cells from *mpeg1:GFP* larvae.

Figure S2

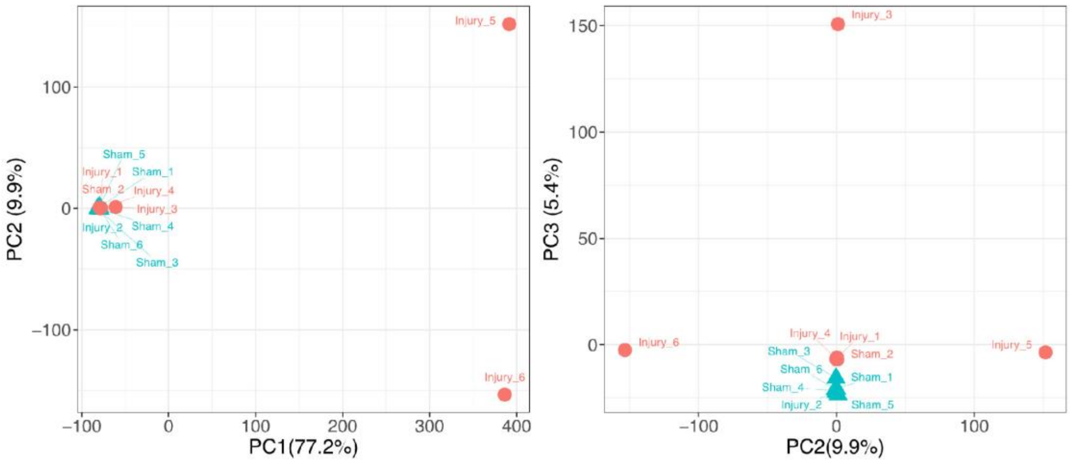

Fig. S2. Principal component analysis reveals clustering patterns of RNA-seq samples. Plots of the first, second and third components from principal component analysis of all 12 RNA-seq samples. Three samples from the 2 hpi experimental group (Injury_3, Injury_5 and Injury_6) displayed high duplication and low mapping rates, and did not cluster well with the other samples.

Biology Open • Supplementary information

Figure S3

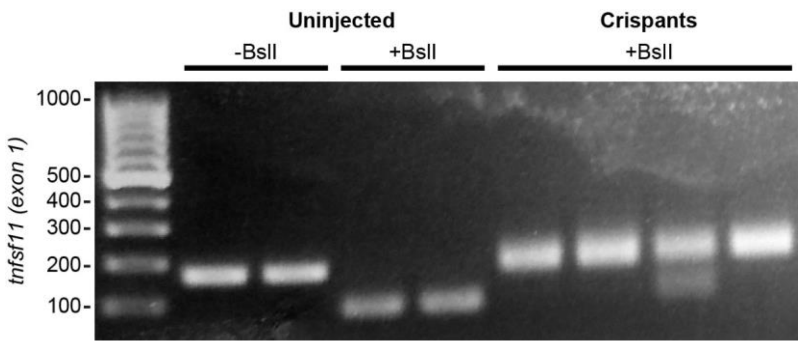

Fig. S3. CRISPR/Cas9-mediated gene editing leads to efficient somatic mutation of the gRNA target site. Restriction fragment length polymorphism analysis of embryos injected with gRNA targeting a BslI restriction site in exon 1 of *tnfsf11*. Somatic mutation of the gRNA target site is induced with an efficiency of 90-100%, as shown by resistance to BslI restriction endonuclease digestion in gRNA-injected embryos. One embryo was analysed per well.

Biology Open • Supplementary information

Figure S4

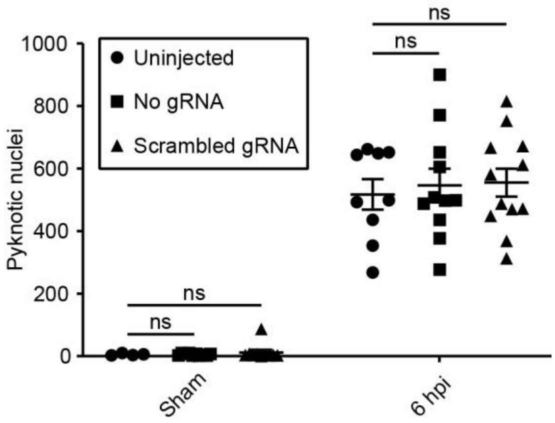

Fig. S4. Injection of scrambled gRNA does not change the extent of secondary cell death. Quantification of pyknotic nuclei within the tectum of uninjected animals, or of animals injected with no gRNA or a scrambled gRNA. n (sham, uninjected) = 4 animals. n (sham, no gRNA) = 10 animals. n (sham, scrambled gRNA) = 10 animals. n (6 hpi, uninjected) = 9 animals. n (6 hpi, no gRNA) = 11 animals. n (6 hpi, scrambled gRNA) = 12 animals. N = 2 independent experiments. Two-way ANOVA with Bonferroni correction was used to compare experimental groups. $p > 0.999$ for sham, uninjected vs sham, no gRNA. $p > 0.999$ for sham, uninjected vs sham, scrambled gRNA. $p > 0.999$ for 6 hpi, uninjected vs 6 hpi, no gRNA. $p > 0.999$ for 6 hpi, uninjected vs 6 hpi, scrambled gRNA. ns, not significant.

Biology Open • Supplementary information

Table S1. Efficiencies of gRNAs.

Gene	Target exon	Restriction enzyme	Target sequence	Efficiency
tnfsf11	exon 1	BslI	CCGGCGCGCGCTCCACACAC	90%
tnfsf11	exon 4	BstNI	TTAACCCAGATAGCCTGGGA	80%
odc1	exon 4	BsrI	GGCTCCCAGAGACGCCAGTG	90%
odc1	exon 10	BslI	CGACGGTCTGGACCGCATTG	100%
smox	exon 3	BslI	CCCCGTCTACCACCTGGCTG	100%

Biology Open • Supplementary information

Table S2. Assessment of reliability of cell death scoring.

**Compound**	**Cell death scoring result**	**Cell counting results**		**Scoring result confirmed?**
		**DMSO**	**Compound**	
Imiquimod	Increased	511 ± 42 (n = 12 larvae)	520 ± 47 (n = 12 larvae)	No
Lindane	Increased	478 ± 46 (n = 10 larvae)	640 ± 50 (n = 10 larvae)	Yes
Tiagabine	Decreased	479 ± 21 (n = 11 larvae)	439 ± 48 (n = 12 larvae)	Yes
Levothyroxine	Decreased	478 ± 46 (n = 10 larvae)	479 ± 49 (n = 9 larvae)	No

The extent of cell death was assessed through scoring, and the number of pyknotic nuclei at 6 hpi was then quantified through manual counting in the same larvae. The scoring result was considered to have been confirmed by counting if the number of pyknotic nuclei in drug-treated larvae differed from that in DMSO-treated larvae by more than 5% in the direction indicated by the scoring result. Despite showing a reliable increase in cell death in injured larvae, lindane was not considered a hit compound because it also increased cell death in sham larvae. Note that the number of pyknotic nuclei in DMSO-treated larvae is the same for lindane and levothyroxine since these compounds were processed in the same experiment.

Biology Open • Supplementary information

Table S3. Primers for RT-qPCR.

Gene	Sequence (5'-3')	Product length	Optimal concentration	E value (efficiency)
tnfsf11	fw: GGCGTTCATGGCAGCTAATG<br>rev: GAGGCAAGATCGACCTCTCG	207 bp	0.5 µM	2.5
odc1	fw: CACGAAAATGCCAAGCTGGT<br>rev: CTGGCTGTATGTTTCCGGGT	195 bp	0.2 µM	2.13
smox	fw: CCTCAAGGTGGAGAGCTGTG<br>rev: CAGGCTTGCTAAGGTGGAGG	170 bp	0.2 µM	2.13

Biology Open • Supplementary information

Table S4. Primers for testing of gRNA efficiencies.

Gene	Target exon	Sequence (5’-3’)	Product length
tnfsf11	exon 1	fw: CGTTCATGGCAGCTAATGAT<br>rev: GCAACAGAATCGCCACACTA	168 bp
tnfsf11	exon 4	fw: AACGCAGTGGTTAAAACAACAA<br>rev: GGGAATCCACTATAATTCGGTTA	244 bp
odc1	exon 4	fw: CTTCTATGTGGCGGATCTGG<br>rev: ACGCATGAAGTGACGGTGTA	204 bp
odc1	exon 10	fw: CGTTCCATTTGAGAACGTGA<br>rev: GTTCTCGAACAGCAGCCAGT	277 bp
smox	exon 3	fw: TCTGCTTCTTTTCAATGTTCC<br>rev: CTCTTCCACCAGATCCTTCG	247 bp

Biology Open • Supplementary information
